# The role of ultra-processed food consumption and depression on type 2 diabetes incidence: a prospective community study in Quebec, Canada

**DOI:** 10.1017/S1368980022002373

**Published:** 2023-11

**Authors:** Akankasha Sen, Anne-Sophie Brazeau, Sonya Deschênes, Hugo Ramiro Melgar-Quiñonez, Norbert Schmitz

**Affiliations:** 1 School of Human Nutrition, McGill University, Sainte-Anne-de-Bellevue, QC, Canada; 2 Douglas Mental Health University Institute, Bd LaSalle, QC, Canada; 3 UCD School of Psychology, University College Dublin, Belfield, Dublin, Ireland; 4 Department of Psychiatry, McGill University, West Montreal, QC, Canada; 5 Department of Population-Based Medicine, Tuebingen University, Hoppe-Seyler-Street 9, Tuebingen 72076, Germany

**Keywords:** Ultra-processed food, Depression, CARTaGENE, Type 2 diabetes

## Abstract

**Objectives:**

The goal of the present study was to evaluate the association between depression and ultra-processed food (UPF) consumption as risk factors for developing type 2 diabetes (T2D).

**Design:**

A prospective community study.

**Setting:**

Baseline data (2009–2010) from CARTaGENE community health study from Quebec, Canada, were used. Food and drink consumption was assessed using the Canadian-Diet History Questionnaire II and grouped according to their degree of processing by the NOVA classification, and participants were categorised into tertiles of UPF (g/d). Depression was defined using either a validated cut-off score on the Patient Health Questionnaire-9 or antidepressant use. The outcome was the incidence of T2D, examined in 3880 participants by linking survey data with administrative health insurance data. Cox regression models estimated the associations between UPF, depression and incident T2D.

**Participants:**

40–69-year-old individuals at baseline.

**Results:**

In total, 263 (6·8 %) individuals developed T2D. Participants with high depressive symptoms and high UPF consumption showed the highest risk for T2D (adjusted hazard ratios (aHR) = 1·58, 95 % CI (0·98, 2·68)), compared to those with low depressive symptoms and low UPF consumption. The risk for T2D was similar when high depressive symptoms and antidepressant use were combined with high UPF (aHR 1·62, 95 % CI (1·02, 2·57)).

**Conclusions:**

This study shows that co-occurring depression and high UPF consumption were associated with a higher risk for T2D. Early management and monitoring of both risk factors might be essential for diabetes prevention.

Type 2 diabetes (T2D) is a worldwide, increasingly prevalent chronic disease that can lead to adverse outcomes, such as microvascular and macrovascular complications, disability and early mortality^(1)^.

Mental health problems, such as depression, are well-established comorbidities of T2D^(2)^. Evidence from meta-analyses has shown that depression increases the risk for T2D incidence by 40–60 %^(2,3)^. The underlying mechanisms explaining this relationship might be multifactorial; it is likely that depression may influence the incidence and consequences of diabetes through behavioural and biological pathways. For example, several lifestyle-related behaviours, such as poor dietary habits and decreased physical activity, can contribute to this relationship^(4,5)^. Therefore, health behaviours are important factors for diabetes risk.

Healthy diets, among other factors such as physical activity, are potentially modifiable factors that can help prevent and manage T2D^(6)^. A meta-analysis of prospective studies found that healthy dietary patterns or healthy diet indexes, such as the Mediterranean, the Dietary Approaches to Stop Hypertension, the Healthy Eating Index and the Alternative Healthy Eating Index, are associated with a lower risk for T2D^(7)^.

The modern food system is facing a considerable challenge due to the rapid increase in the availability and consumption of ultra-processed foods (UPF) and drinks^(8)^. UPF are defined as formulations of industrial ingredients that result from a series of industrial processes (hence ‘ultra-processed’). They typically are of low nutritional quality and contain little or no whole foods, are ready-to-consume or heat up and are fatty, salty or sugary and depleted in dietary fibre, protein, various micronutrients and other bioactive compounds^(8)^. Recent studies from high-income countries, including Canada, suggest that UPF account for 50–60 % of the total daily energy intake^(9–11)^. Higher consumption of UPF was associated with a 31 % increased risk of obesity, a 37 % increased risk of diabetes and a 60 % increased risk of hypertension^(12)^.

Although depression and UPF consumption have independently been shown to increase the risk for T2D^(4,12)^, the extent to which the combination of these factors increases the risk for T2D has yet to be investigated. For example, depressive symptoms are associated with high levels of inflammatory markers such as C-reactive protein, TNF-*α*, IL-1 and IL-6 levels, which are associated with T2D^(13)^. Further, UPF are also associated with these inflammatory markers^(14)^ and are also associated with T2D incidence^(12)^. Therefore, it is possible that depression and UPF consumption may exacerbate a common pathway, resulting in a substantially elevated risk for T2D incidence. Hence, it is particularly relevant to increase our understanding of the relationship between UPF consumption, depression and the risk of T2D.

Thus, the goal of this study was to prospectively investigate the potential additive interaction between UPF consumption and depression on the incidence of T2D in a Canadian community sample. We hypothesised that individuals with both high depression and high UPF consumption at baseline would have a higher risk of developing T2D than those with high depression only and with high UPF consumption only or neither.

## Materials and methods

### Study population

Baseline data used in this study were from the CARTaGENE (www.cartagene.qc.ca) cohort study (2009–2010), a community health survey conducted in the Canadian province of Quebec in the adult population aged 40–69 years living in metropolitan areas (Montreal, Quebec City, Sherbrooke and Saguenay)^(15)^. CARTaGENE participants were randomly recruited from the governmental provincial health insurance database, the Régie de l’Assurance Maladie du Québec (RAMQ). Under this government health insurance plan, most residents of Quebec have health coverage^(15)^. Recruitment, enrolment and data collection methods are described in detail elsewhere^(15)^. All participants provided informed consent to participate in the CARTaGENE cohort study and agreed to have their data linked with the provincial health insurance database. Participants provided information on demographic, health characteristics and biospecimens for clinical measures during their interviews^(15)^. Additionally, a nutrition component was added to a subset of the participants^(15)^. This component includes a questionnaire relating to eating habits (described below). Follow-up data referring to T2D incidence were obtained by linking participants with diagnostic codes from the RAMQ database.

### Measures

#### Depressive symptoms

Depressive symptoms were measured using the Patient Health Questionnaire-9, which consists of nine questions related to vegetative, emotional, behavioural and cognitive symptoms during the past 2 weeks^(16)^. Responses ranged from ‘not at all’ (0) to ‘every day’ (3), with a summary score ranging from 0 to 27. Depressive symptoms were defined as having a Patient Health Questionnaire-9 summary score of 6 and higher, which includes moderate to severe depressive symptoms. In this study, a score of 6 or higher is categorised as ‘high depressive symptoms’. This cut-off score has shown good performance and has been used in many studies, including CARTaGENE cohort^(17,18)^. When compared with the fully structured interviews for major depressive disorder, the Patient Health Questionnaire-9 cut-off of 6 has a sensitivity of 0·91 and a specificity of 0·61^(19)^.

#### Antidepressant use

Participants brought their current medication or reported their current medication at the baseline interview. Medication was classified as an antidepressant based on the medication name^(20)^.

#### Dietary intake assessment

Dietary intake in the CARTaGENE survey was measured using the Canadian-adapted Diet History Questionnaire II (C-DHQ II)^(21)^. C-DHQ II is a FFQ initially developed by the US National Cancer Institute and modified to reflect food availability, brand names, nutrition composition and food fortification in Canada^(22)^. It contains 164 questions related to food, portion size, frequency and vitamin/mineral supplement use during the last 12 months.

A commonly used unit or portion size is specified for most food items. Daily consumption of each FFQ food item was computed based on one of four units of time, depending on which answer choice was selected: year, month, week or day. For this study, all the items used for the analysis were using ‘all year’ format, which implies that items using daily consumption for summer, not in summer, winter, not in winter, in season and out of season format were not included in the present study^(21,23)^.

Daily consumption of the items was converted into daily equivalents such as never = 0; 1–6/year = 0·01; 7–11/year = 0·02; 1/month = 0·03; 2–3/month = 0·07; 1/week = 0·14; 2/week = 0·29; 3–4/week = 0·48; 5–6/week = 0·74; 1/day = 1; 2 or more = 3 as specified by the C-DHQII database^(23)^. Secondly, portions of consumed food items were converted into grams by using the nutrient database for the C-DHQII^(24)^. Portions are sex-specific and based on the percentiles of intake reported in the Canadian Community Health Survey – Cycle 2.2 Nutrition^(22)^. Then, the consumed amount for every food item was calculated by multiplying the frequency per day and grams of consumption. In the present analysis, food items without portion size and items such as vitamins, minerals or herbal supplements were excluded.

Similar to any lengthy and self-administered questionnaire, FFQ is often associated with non-responses. Food items on an FFQ might be omitted for different reasons, for example, the food may not be consumed by respondents or they might have difficulties remembering the frequency and amount of intake^(25,26)^. Therefore, zero imputation was used to deal with missing data in FFQ based on the assumption that items which were left blank in the data were not consumed by the respondent^(26)^.

Food and beverage items of FFQ were categorised according to NOVA (not an acronym) food groups – a classification system which considered all physical, biological and chemical modification that occurs to foods after they are separated from their natural form^(8)^. As a result, all foods are classified into one of four groups. NOVA group 1 includes unprocessed or minimally processed foods, meaning foods processed in a way that does not add or introduce a substance to the original food. However, these foods might involve processing with the aim of extending the shelf-life of unprocessed foods, allowing their storage for longer use and facilitating or diversifying food preparation. Fruit and vegetables, grains (cereals), fresh and pasteurised milk products and meat and fish are some examples of NOVA group 1. NOVA group 2 comprises processed culinary ingredients such as salt, sugar, vegetable oil and butter. These products are extracted and refined from NOVA group 1 food or obtained from nature. Pressing, refining, grinding, mining and spray drying are the methods involved in obtaining these products. NOVA group 3 contains processed foods to which salt, sugar or other substances of culinary use, such as oil or vinegar, have been added, and methods involving smoking, curing or fermentation have been performed to preserve them or to enhance their palatability. Food products in this group are canned or bottled food items such as vegetables and fruits, cheeses and freshly made bread. NOVA group 4 comprises UPF and drinks that were prepared mostly or entirely from substances derived from industrial foods, with little or no whole food content. Ingredients present in these foods are modified starches, hydrogenated oils, protein isolates and additives whose purpose is to increase the shelf life, hyper-palatable, protect original properties and prevent the proliferation of micro-organisms. Examples of products are ready-to-eat meals, carbonated drinks, biscuits, processed meat, and sugared milk and fruit drinks^(8)^. Food and beverage items that were defined in the fourth category of the NOVA classification for the present study were identified and verified by the two researchers, and these UPF items were also classified in group 4 in the other cohorts, such as Nurses’ Health Studies, The Health Professionals Follow-up Study and Growing Up Today Studies^(27)^.

To estimate UPF consumption (g/d), we summed the amount consumed (g/d) of each food and beverage item classified in the fourth category of the NOVA classification (a total of thirty foods and seven beverage items). Participants were then divided into tertiles according to the total consumption of UPF (g/d). Low and middle tertiles were considered as one group for joint association analysis.

#### Incidence of type 2 diabetes mellitus

The primary outcome was the incidence of diabetes. This was assessed using diagnostic codes in RAMQ billing database. Diagnostic codes were based on the World Health Organization’s International Classification of Diseases, 9th, or 10th edition (ICD-9 and ICD-10, respectively), and the code was ICD-9 code 250 and ICD-10 code E11. Participants were followed for a maximum of 7 years using administrative data from the date of their CARTaGENE baseline assessment. The date of the first diagnosis or hospital admission for diabetes was recorded. Observational time was calculated from the day of baseline assessment to the day of T2D onset, the date of death or the study end date of 31 December 2016.

#### Covariates

Several factors might affect the association between depression, UPF consumption and T2D incidence. We, therefore, included the following covariates in our analyses: age, sex, self-reported ethnicity (white and other), marital status, education, annual household income and country of birth (born in Canada or outside) and smoking status (‘currently smokes daily or occasionally’, ‘past smoker’ or has ‘never smoked’). Alcohol consumption was defined as whether participants consumed alcohol daily or not. Physical activity was measured by asking participants ‘how many days in the last week they engaged in moderate/vigorous activity’. High physical activity was defined as 5 or more days with moderate activity or 3 or more days with vigorous activity in the past week.

### Statistical analysis

#### Inclusion criteria

**Fig. 1:**
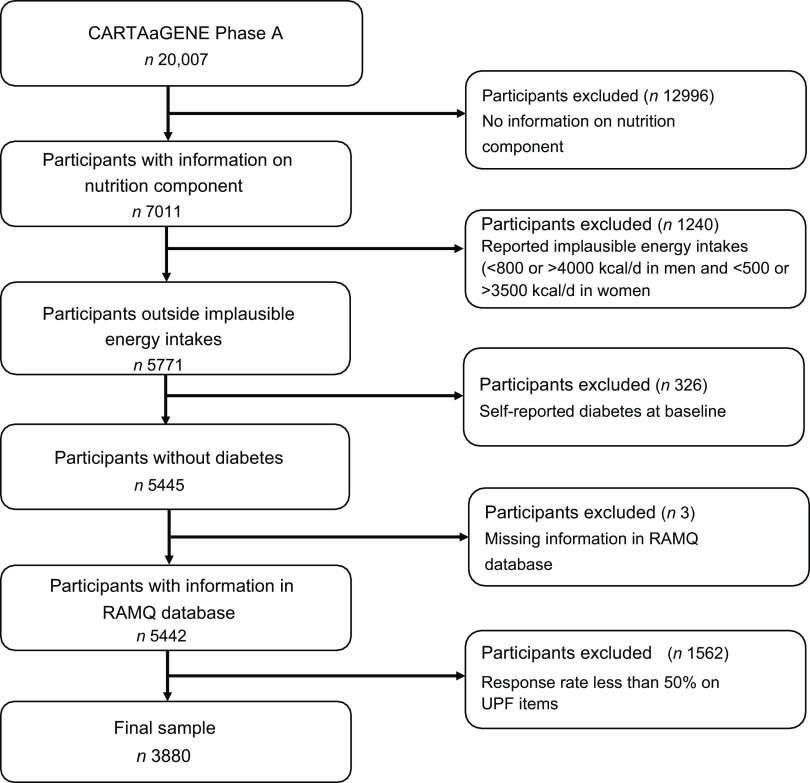
Flow diagram of the final sample for the analysis. RAMQ, Régie de l’Assurance Maladie du Québec; UPF, ultra-processed foods

Only participants with information on the nutrition component at baseline were included (*n* 7011). Implausible reporting, particularly under-reporting, is a widely recognised limitation of dietary assessment methods; participants tend to underestimate their total energy intakes and under-report intakes of foods that are deemed unhealthy or socially undesirable, such as foods that are high in fat and refined carbohydrates. Therefore, we excluded all participants using the simpler recommended method (excluding implausible energy intakes below 800 kcal/d or above 4000 kcal/d in men and below 500 kcal/d or above 3500 kcal/d in women (*n* 1240)). This approach has been used in previous studies^(28)^. There were more under-reporter (*n* 992) than over-reporter (*n* 248) in our sample. Further, those participants who reported diabetes at baseline based on a positive response to the following question: ‘Has a doctor ever told you that you had diabetes?’ (*n* 326) and those whose data could not be linked to the provincial health insurance database (*n* 3) were also excluded. In addition, participants whose response rates were less than 50 % on the UPF items (*n* 1562) were excluded. A total of 3880 participants were included in the final analyses (Fig. 1). Moreover, we performed two sensitivity analyses first with a) a 40 % response rate on UPF items (sample size *n* 4364) and b) a 60 % response rate on the UPF items (sample size *n* 3012) to test the robustness of the study (online supplementary data).

Cox proportional hazards regression models were first conducted to examine the individual association of UPF consumption, depression symptoms and antidepressant use on the incident T2D. To evaluate a potential additive interaction between UPF and depressive symptoms, we defined four groups: (a) lower/middle tertile of UPF consumption and low depressive symptoms (LUND as the reference group); (b) lower/middle tertile of UPF consumption and high depressive symptoms (LUD); (c) higher tertile of UPF consumption and low depressive symptoms (HUND) and (d) higher tertile of UPF consumption and high depressive symptoms (HUD). Cox regression was conducted to evaluate a potential additive interaction of depressive symptoms and UPF on T2D incidence. Finally, an additional analysis was performed by combining depressive symptoms with antidepressant medications as an indicator of depression. Four groups were created: (a) lower and middle tertile of UPF consumption and low depressive symptoms and no antidepressant use (LUNDA as the reference group); (b) lower and middle tertile of UPF consumption and high depressive symptoms or antidepressant use (LUDA); (c) higher tertile of UPF consumption and low depressive symptoms and no antidepressant use (HUNDA) and (d) higher tertile of UPF consumption and high depressive symptoms or antidepressant use (HUDA).

All the Cox regression analyses were performed in unadjusted models, in models adjusted for age and sex only and in fully adjusted models. Hazard ratios (HR) with 95 % CI are reported. Missing information on the covariates was imputed using the fully conditional specification with discriminant or logistic methods using PROC MI procedure SAS. To examine the interaction between depression and UPF consumption on the risk of T2D development, the RERI (relative excess rate due to interaction) index was computed^(29)^. RERI is an index for an interaction on the additive scale and was calculated using the following equation: RERI = HR_AB_ – HR _Ab_– HR_aB_ + 1^(29)^, where HR_AB_ is the presence of both elevated depression and UPF consumption, HR_Ab_ is the presence of depression only and HR_aB_ is the presence of UPF consumption only. A RERI greater than zero indicates a more than additive (synergistic) interaction^(29)^.

## Results

The main food groups contributors to UPF intake are shown in Table 1. Overall mean consumption of the UPF was 225·8 g/d (sd 331·8), and mean consumption in the lower, middle and highest tertile was 107·1 (sd 33·9), 209·1 (sd 33·4) and 579·5 g/d (sd 407·0), respectively. Soft and isotonic drinks, fast food and ready to eat and cookies, biscuits, muffins and cake food groups were the main food groups contributing to the total of UPF.

**Table 1:** Contribution of each food group to the total amount of ultra-processed foods consumed in the CARTaGENE study cohort (*n* 3880)

Food groups (*n* 37)	Contribution to total ultra-processed foods intake (%)*	Daily amount consumed mean g/d	sd
Beverages (*n* 7)			
Dairy beverages	5·3	11·8	38·3
Soft/isotonic drinks	44·0	99·3	294·6
Fruit drinks	4·5	10·2	87·4
Solid foods (*n* 30)			
Processed meat	4·5	10·2	15·2
Fast food and ready to eat	11·2	25·4	27·9
Breakfast cereals	4·5	10·1	14·5
Cookies, biscuits, muffins and cake	11·7	26·5	37·7
Potato chips and salty snacks	3·4	7·6	10·5
Confectionery and chocolate	2·8	6·3	15·3
Ketchup, salad dressing and similar	4·5	10·2	12·3
Ice-cream	2·3	5·2	10·6
Jelly and jams products	1·4	3·1	6·1
Total	100	225·8	331·8

* Contribution (%) of each food group/beverage to the total consumption of ultra-processed food was calculated by dividing the amount (g/d) of each food group by the total amount of ultra-processed foods (g/d) multiplied by 100.

Sample characteristics are presented in Table 2. At baseline, the sample was on average 54·2 years old (sd = 7·5). There were 2327 (60 %) participants in the LUND group (reference group); 260 (6·7 %) participants in the LUD group; 1114 (28·7 %) participants in the HUND group and 179 (4·6 %) participants in the HUD group. Participants in the HUD group had a higher mean intake of UPF: 605 (711·5) g/d. Compared with participants in the other groups, they were more likely to be smokers, physically inactive and have a lower proportion of post-secondary education. A total of 263 (6·8 %) individuals developed T2D during the observation period. T2D incidence was 5·9, 6·9, 8·2 and 8·9 % for LUND, LUD, HUND and HUD, respectively.

**Table 2:** Baseline characteristics of the study sample

	LUND (*n* 2327)		LUD (*n* 260)		HUND (*n* 1114)		HUD (*n* 179)		Total (*n* 3880)	
	Mean	sd	Mean	sd	Mean	sd	Mean	sd	Mean	sd
Age	55·0	7·7	53·7	6·8	53·2	7·4	52·0	7·4	54·2	7·5
	*n*	%	*n*	%	*n*	%	*n*	%	*n*	%
Sex										
Male	750	32·2	66	25·4	656	58·9	84	46·9	1556	40·1
Female	1577	67·8	194	74·6	458	41·1	95	53·1	2324	59·9
Household income										
Lower income level (<49 999 $)	631	27·1	110	42·3	298	26·8	67	37·4	1106	28·5
Middle income level (50 000–149 999 $)	1429	61·4	132	50·8	694	62·3	103	57·5	2358	60·8
High income level (>150 000 $)	267	11·5	18	6·9	122	11·0	9	5·0	416	10·7
Post-secondary education										
No	441	19·0	72	27·7	275	24·7	58	32·4	846	21·8
Yes	1886	81·0	188	72·3	839	75·3	121	67·6	3034	78·2
Born in Canada										
No	241	10·4	39	15·0	94	8·4	23	12·8	397	10·2
Yes	2086	89·6	221	85·0	1020	91·6	156	87·2	3483	89·8
Ethnicity										
Other	170	7·3	25	9·6	63	5·7	13	7·3	271	7·0
White	2157	92·7	235	90·4	1051	94·3	166	92·7	3609	93·0
Marital status										
Married/partner	1583	68·0	131	50·4	778	69·8	103	57·5	2595	66·9
Single	277	11·9	53	20·4	158	14·2	39	21·8	527	13·6
Divorced/separated/widowed	467	20·1	76	29·2	178	16·0	37	20·7	758	19·7
Daily alcohol consumption										
No	2020	86·8	234	90·0	993	89·1	168	93·9	3415	88·0
Yes	307	13·2	26	10·0	121	10·9	11	6·1	465	12·0
Smoking status										
Daily and occasional	283	12·2	53	20·4	203	18·2	42	23·5	581	15·0
Past smoking	988	42·4	102	39·2	447	40·1	60	33·5	1597	41·2
Never smoking	1056	45·4	105	30·4	464	41·7	77	43·0	1702	43·9
Physical activity										
No	1405	60·4	182	70·0	646	58·0	126	70·4	2359	60·8
Yes	922	39·6	78	30·0	468	42·0	53	29·6	1521	39·2
Diabetes incidence										
No	2189	94·1	242	93·1	1023	91·8	163	91·1	3617	93·2
Yes	138	5·9	18	6·9	91	8·2	16	8·9	263	6·8
	Mean	sd	Mean	sd	Mean	sd	Mean	sd	Mean	sd
UPF consumption g/d	102·9	43·7	106·1	46·2	449·4	436·1	605·4	711·5	225·8	331·8

LUND, lower/middle tertile of ultra-processed foods consumption and low depressive symptoms; LUD, lower/middle tertile of ultra-processed foods consumption and high depressive symptoms; HUND, higher tertile of ultra-processed foods consumption and low depressive symptoms; HUD, higher tertile of ultra-processed foods consumption and high depressive symptoms; UPF, ultra-processed foods.

When compared with the complete baseline CARTaGENE sample (without diabetes), our sample included a greater proportion of females (59·9 % compared with 47·6 % of the overall baseline population) and a greater proportion of participants in the middle-income level compared to the baseline sample (60·8 and 50·3 %, respectively).

Table 3 describes the results of three univariate Cox regression analyses. Participants in the highest tertile of UPF consumption had the highest risk for T2D incidence in the fully adjusted model (HR = 1·47, 95 % CI (1·07, 2·03)) as compared to those with the lowest UPF consumption. The HR for depressive symptoms (Patient Health Questionnaire-9 ≥ 6) was 1·12 (95 % CI (0·85, 1·76)) when adjusted for all the covariates. Similarly, HR for antidepressant use was 1·31 (95 % CI (0·85, 2·01)) in the fully adjusted model.

**Table 3:** Results of Cox regression for UPF consumption and depression assessed using PHQ-9 and antidepressant for incident T2D

Groups	*n*	Incident T2D	Unadjusted model, HR	95 % CI	Age- and sex-adjusted model, HR	95 % CI	Fully adjusted model, HR	95 % CI*
Model 1: UPF consumption univariate association								
Lower tertile of UPF consumption	1293	70	Reference		Reference		Reference	Reference
Middle tertile of UPF consumption	1294	86	1·23	0·90, 1·69	1·20	0·87, 1·66	1·26	0·91, 1·74
Higher tertile of UPF consumption	1293	107	1·55	1·15, 2·10	1·50	1·09, 2·07	1·47	1·07, 2·03
Model 2: Depression univariate association								
PHQ-9 summary score (< 6) Low	3441	229	Reference		Reference		Reference	Reference
PHQ-9 summary score (>= 6) High	439	34	1·18	0·82, 1·69	1·34	0·93, 1·93	1·22	0·85, 1·76
Model 3: Antidepressant use univariate association								
Antidepressant use NO	3599	240	Reference		Reference		Reference	Reference
Antidepressant use YES	281	23	1·25	0·81, 1·91	1·39	0·90, 2·14	1·31	0·85, 2·01

UPF, ultra-processed foods; PHQ-9, patient health questionnaire-9; T2D, type 2 diabetes; HR, hazard ratio.

* Fully adjusted model is adjusted for the following variables: age, sex, household income, education, ethnicity, born in Canada, smoking status, physical activity and daily alcohol consumption.

Table 4 presents the results from the additive interaction analysis, and the reference category in model 1 was the LUND group. Participants in the HUD group had the highest risk of T2D: the HR was 1·58 (95 % CI (0·93, 2·68)) in models adjusted for all the covariates. Those in the HUND group had a higher risk for T2D compared with those in the LUD group. The RERI coefficient was 0·26 (95 % CI (-0·32, 1·45)) in the adjusted model, suggesting a more than additive interaction. However, the CI is wide and includes 0. We found a similar risk in model 2 when combining depressive symptoms and antidepressant medication as indicators for depression. The highest risk for T2D was found in the HUDA group in the model adjusted for age and sex (HR 1·78, 95 % CI (1·13, 2·81)) and in the model adjusted for all the covariates (HR 1·55, 95 % CI (1·01, 2·37)). The RERI was 0·09 (95 % CI (-0·81, 1·23)) in the adjusted model. Our sensitivity analyses showed similar results, suggesting that participants with both conditions, depression and UPF consumption, were at higher risk for developing diabetes than their counterparts (online supplementary Data).

**Table 4:** Results of Cox regression for UPF consumption and depression assessed using PHQ-9 and antidepressant joint association for incident T2D

Groups	*n*	Incident T2D (*n*)	Unadjusted, HR	95 % CI	Age- and sex-adjusted model, HR	95 % CI	Fully adjusted model, HR*	95 % CI
Model 1 UPF consumption lower and middle tertile combined and depressive symptoms joint association								
LUND	2327	138	Reference		Reference		Reference	
LUD	260	18	1·17	0·72, 1·92	1·32	0·80, 2·15	1·21	0·73, 1·98
HUND	1114	91	1·39	1·07, 1·81	1·34	1·02, 1·77	1·28	0·97, 1·69
HUD	179	16	1·56	0·93, 2·62	1·75	1·04, 2·95	1·58	0·93, 2·68
Model 2 UPF consumption lower and middle tertile combined and depressive symptoms/antidepressant use joint association								
LUNDA	2207	127	Reference		Reference		Reference	
LUDA	380	29	1·34	0·89, 2·01	1·49	0·99, 2·23	1·38	0·92, 2·07
HUNDA	1046	85	1·43	1·08, 1. 88	1·37	1·03, 1·82	1·31	0·98, 1·74
HUDA	247	22	1·60	1·02, 2·51	1·78	1·13, 2·81	1·62	1·02, 2·57

LUND, lower/middle tertile of ultra-processed foods consumption and low depressive symptoms; LUD, lower/middle tertile of ultra-processed foods consumption and high depressive symptoms; HUND, higher tertile of ultra-processed foods consumption and low depressive symptoms; HUD, higher tertile of ultra-processed foods consumption and high depressive symptoms; LUNDA, lower and middle tertile of ultra-processed foods consumption and low depressive symptoms and no antidepressant use; LUDA, lower and middle tertile of ultra-processed foods consumption and high depressive symptoms or antidepressant use; HUNDA, higher tertile of ultra-processed foods consumption and low depressive symptoms and no antidepressant; HUDA, higher tertile of ultra-processed foods consumption and high depressive symptoms or antidepressant; T2D, type 2 diabetes; HR, hazard ratio.

* Fully adjusted model is adjusted for the following variables: age, sex, household income, education, ethnicity, born in Canada, smoking status, physical activity and daily alcohol consumption.

## Discussion

In this prospective community study of 3880 individuals aged 40–69 years without T2D at baseline, we evaluated the impact of depression and UPF consumption on T2D incidence over approximately 7 years. The results suggest an interaction between depression and UPF consumption in relation to an increased risk of T2D. Cox regression analyses indicated that participants with both elevated depression and high UPF consumption at baseline were at an increased risk of developing T2D compared to those with high depression only and those with high UPF consumption only or neither.

To our knowledge, no prior study evaluated the interaction of UPF consumption and depressive symptoms on T2D incidence. Previous studies have looked at interactions between depression and metabolic factors on T2D incidence. One study has reported that the interaction between depression and obesity was more strongly associated with the risk of T2D than the sum of the individual effect^(30)^. Further, a similar finding was seen in Midlife Development Study in the USA, where interactions were found between central obesity and depression on T2D incidence (adjusted risk ratios = 2·16, 95 % CI (1·18, 3·98))^(31)^.

A Canadian study found that the combined effect of depressive symptoms and metabolic dysregulation increased the risk of T2D over a 4-year follow-up period (adjusted OR = 6·61, 95 % CI (4·86, 9·01))^(17)^. A cross-sectional study from Australia (13 763 men aged 18–55 years) showed that men having both comorbid depression and obesity had a 7·6 (OR) times higher risk of T2D compared to men without comorbid depression and obesity^(32)^.

There are several pathways in which depression or depressive symptoms may be associated with an increased risk of developing T2D. Health-risk behaviours might have a key role in this association^(3)^. A meta-analysis of longitudinal studies examining depression as a risk factor for diabetes found that adjustment in BMI and lifestyle factors (mainly physical activity) lowered the risk of T2D in people with depression, suggesting that higher BMI and physical inactivity might contribute to the association discussed above^(2)^. Healthy behaviours, including good eating behaviours, are significant lifestyle factors that can lower the risk of T2D. Depression has been shown to adversely impact these behaviours^(2,3)^, which might affect the management of T2D. Depression is associated with increased caloric consumption and less involvement in physical activity^(33,34)^, which can be related to an increase in weight and an increase in T2D risk^(35)^.

Higher consumption of UPF and depressive symptoms share common biological mechanisms, and the co-occurrence of both conditions might intensify the risk of developing T2D. First, activation of the hypothalamic–pituitary–adrenocortical (HPA) axis and the autonomic nervous system might play a key role. Unstable cortisol concentrations are linked with depression, obesity, insulin resistance and T2D^(3)^. Depressive symptoms and T2D are linked with the hypothalamic–pituitary–adrenocortical axis in disease development. Obesity, which is linked with higher consumption of UPF^(12)^, is a well-established risk factor development of T2D^(12)^. Further, a high concentration of inflammatory markers may be involved in developing T2D in individuals with depression^(13)^. Studies have reported that both depressive symptoms and UPF or western-style dietary patterns are associated with inflammatory markers such as C-reactive protein, TNF-*α*, IL-1 and IL-6 levels^(13,14)^.

Additionally, diets high in sugar, commonly found in UPF^(36)^, might be a potential mechanism mediating the relationship between depressive symptoms and T2D. High sugar consumption can activate brain regions associated with the reward response and provoke a more intense feeling of hunger than in low-sugar diets^(37)^. These reward responses can drive the loss of self-control, overeating and subsequent weight gain, leading to the development of T2D^(37,38)^. Furthermore, consuming sweet foods and added sugar has also been linked to depression^(39)^.

Some antidepressant medications might act as mediators of this relationship since antidepressant use seems to be associated with long-term weight gain (for some antidepressants) and may represent a key biological factor for the development of T2D^(13,40)^. Higher UPF consumptions and depressive symptoms might stimulate each other’s occurrence, which can, in turn, result in obesity, inflammation and insulin resistance. Therefore, it is possible that it can lead to a vicious cycle that further increases the risk of depression and T2D.

Furthermore, beyond the unhealthy nutritional composition of UPF, these foods are also impacting health in different pathways. Recent concern has emerged about the manufacturing and packaging process of UPF. Studies have linked the cosmetic additives commonly used in UPF, such as flavours, emulsifiers and thickeners, to gut dysbiosis and may initiate inflammation in the gut^(41)^. Besides these, contamination from food packaging (e.g. phthalates, bisphenol A) is linked to adverse health effects^(42,43)^. However, more research is needed to understand the mechanisms of action and the relative effects of UPF on health.

### Strength and limitation

This study utilised a sample of individuals with no diabetes at baseline and up to 7 years of follow-up data. This study combined survey and administrative data to evaluate the association of UPF and depressive symptoms on T2D incidence in middle-aged individuals. We used two different measures of depression, and the robustness of the study findings was assessed using two different response rates of UPF consumption in a sensitivity analysis.

Our work also has several limitations. Data on diabetes at baseline were based on self-reports and not on clinical measures. Although diabetes surveillance systems in Canada use at least one hospitalisation record or at least two physician claims in a 2-year period, we choose the single claim to diagnose the diabetes cases because of our limited follow-up time. Depressive symptoms were assessed at baseline only. The Patient Health Questionnaire-9 is a self-report scale that measures depressive symptoms experienced in the past 2 weeks and does not account for the history and treatment of depression. Given that depressive symptoms were not measured during the follow-up, symptoms may vary and change over time.

Similarly, dietary intake data measured using C-DHQ II at baseline were assessed by self-report, which may be subjected to reporting bias. Further, dietary intake data were only available at one point in time; therefore, it might be possible that participants change their intake of UPF during the follow-up. Thus, a potential effect of diet quality change over time cannot be established. Participants of the CARTaGENE study were volunteers in a nutrition component and thus are more interested in nutritional issues and healthy lifestyles than the general population. Their consumption of UPF may be lower compared to the general population, which may underestimate the risk investigated in our study. The C-DHQ II was not specifically designed to collect data about the new NOVA classification of UPF consumption. CARTaGENE participants were also limited to mostly white participants and metropolitan; thus, generalisation to other ethnic groups and rural areas cannot be established^(16)^.

## Conclusion

In conclusion, in this large-scale longitudinal study combining survey and administrative data, we evaluated the combined effect of UPF consumption and depression on the incidence of T2D in individuals aged 40–69 years, with up to 7 years of follow-up. This research highlights the interaction between UPF consumption and depressive symptoms as potentially modifiable risk factors for T2D. Given the unprecedented rates of diabetes worldwide, the scientific community needs to do more to understand the risk factors of T2D, and interaction between risk factors may be one approach.

In clinical practice, early management and monitoring of both risk factors might be an important step in the diabetes prevention strategy.

## Supplementary material

For supplementary material accompanying this paper visit https://doi.org/10.1017/S1368980022002373. click here to view supplementary material
