# Supplementary material for: The role of ultra-processed food consumption and depression on type 2 diabetes incidence: a prospective community study in Quebec, Canada
Source: Public Health Nutr. 2022 Nov 4;26(11):2294–303. doi: 10.1017/S1368980022002373 (PMC10641613; doi:10.1017/S1368980022002373)
Supplement: Supplementary file 1 [file S1368980022002373sup001.docx]

The role of ultra-processed food consumption and depression on type 2 diabetes incidence: a prospective community study in Quebec, Canada

Online Supplementary Material

We performed to two sensitivity analysis, by performing the Cox regressions first with a 40% and second with a 60% response rate on the ultra-processed food and beverage items.

1. 40% response rate 15 questions out of 37 on UPF, sample size n = 4365

**Supplementary Table 1.**  Results of cox regression for UPFs consumption and depression assessed using PHQ9 and anti-depressant for incident type 2 diabetes in CARTaGENE

| Groups | N | Unadjusted Model, HR (95% CI) | Age- and Sex-Adjusted Model, HR (95% CI) | Fully Adjusted Model, HR (95% CI) * |
| --- | --- | --- | --- | --- |
| **Model 1: UPFs consumption univariate association** | | | | |
| Lower t**ertile** Of UPFs consumption | 1454 | Reference | Reference | Reference |
| Middle t**ertile** Of UPFs consumption | 1455 | 1.32 (0.99-1.77) | 1.29 (0.96-1.73) | 1.34 (1.00 -1.81) |
| Higher t**ertile** Of UPFs consumption | 1455 | 1.47 (1.11-1.96) | 1.43 (1.06-1.94) | 1.40 (1.03-1.89) |
| **Model 2: Depression univariate association** | | | | |
| PHQ-9 summary score (< 6) Low | 3861 | Reference | Reference | Reference |
| PHQ-9 summary score (>= 6) High | 503 | 1.14 (0.81-1.60) | 1.28 (0.91-1.80) | 1.15 (0.91-1.63) |
| **Model 3: Anti-depressant use univariate association** | | | | |
| Anti-depressant use NO | 4043 | Reference | Reference | Reference |
| Anti-depressant use YES | 321 | 1.22 (0.82- 1.83) | 1.36 (0.94 - 2.08) | 1.30 (0.86 – 1.95) |
| UPFs, Ultra-processed foods; PHQ-9, Patient Health Questionnaire-9.  *Fully adjusted model is adjusted for the following variables: age, sex, household income, education, ethnicity, born in Canada, smoking status, physical activity, daily alcohol consumption. | | | | |

**Supplementary Table 2**  Results of cox regression for UPFs consumption and depression assessed using PHQ9 and anti-depressant joint association for incident type 2 diabetes in CARTaGENE

| **Model 1** **UPFs consumption lower & middle tertile combined and depressive symptoms joint association** | | | | |
| --- | --- | --- | --- | --- |
| **Groups** | **N** | **Unadjusted** | **Age- and Sex-Adjusted Model, HR (95% CI)** | **Fully Adjusted Model, HR (95% CI) *** |
| LUND | 2323 | Reference | Reference | Reference |
| LUD | 264 | 1.00 (0.60 -1.68) | 1.12 (0.66-1.88) | 1.00 (0.59-1.69) |
| HUND | 1118 | 1.31 (1.00 -1.71) | 1.26 (0.95 -1.65) | 1.21 (0.92 -1.59) |
| HUD | 175 | 1.76 (1.07-2.87) | 1.98(1.20-3.24) | 1.80 (1.01-2.98) |
| **Model 2 UPFs consumption lower & middle tertile combined and depressive symptoms/Antidepressant use joint association** | | | | |
| LUNDA | 2205 | Reference | Reference | Reference |
| LUDA | 382 | 1.21 (0.82-1.84) | 1.35 (0.89-2.04) | 1.24 (0.82-1.89) |
| HUNDA | 1048 | 1.34 (1.02-1.77) | 1.28 (0.96-1.70) | 1.23 (0.93-1.66) |
| HUDA | 245 | 1.72 (1.11 -2.65) | 1.92 (1.24 -3.00) | 1.76 (1.13 -2.74) |
| LUND, lower/middle tertile of ultra-processed foods consumption and low depressive symptoms; LUD, lower/middle tertile of ultra-processed foods consumption and high depressive symptoms; HUND, higher tertile of ultra-processed foods consumption and low depressive symptoms; HUD, higher tertile of ultra-processed foods consumption and high depressive symptoms; LUNDA, lower and middle tertile of of ultra-processed foods consumption and low depressive symptoms and no antidepressant use; LUDA, lower and middle tertile of ultra-processed foods consumption and high depressive symptoms or antidepressant use; HUNDA, higher tertile of ultra-processed foods consumption and low depressive symptoms and no antidepressant; HUDA, higher tertile of ultra-processed foods consumption and high depressive symptoms or antidepressant.  *Fully adjusted model is adjusted for the following variables: age, sex, household income, education, ethnicity, born in Canada, smoking status, physical activity, daily alcohol consumption. | | | | |

1. 60% response rate 22 questions out of 37 on UPFs, sample size n = 3012

**Supplementary Table 3**  Results of Cox Regression for UPFs consumption and Depression Assessed Using PHQ9 and Anti-depressant for incident type 2 diabetes in CARTaGENE

| Groups | N | Unadjusted | Age- and Sex-Adjusted Model, HR (95% CI) | Fully Adjusted Model, HR (95% CI) * |
| --- | --- | --- | --- | --- |
| **Model 1: UPFs consumption univariate association** | | | | |
| Lower t**ertile** Of UPFs consumption | 1004 | Reference | Reference | Reference |
| Middle t**ertile** Of UPFs consumption | 1004 | 1.25 (0.88-1.76) | 1.26 (0.89-1.79) | 1.37 (0.96. -1.95) |
| Higher t**ertile** Of UPFs consumption | 1004 | 1.45 (1.03-2.02) | 1.50 (1.06 – 2.12) | 1.52 (1.07-2.17) |
| **Model 2: Depression univariate association** | | | | |
| PHQ-9 summary score (< 6) Low | 2655 | Reference | Reference | Reference |
| PHQ-9 summary score (>= 6) High | 357 | 1.19 (0.80 – 1.76) | 1.32 (0.89 -1.96) | 1.21 (0.81-1.81) |
| **Model 3: Anti-depressant use univariate association** | | | | |
| Anti-depressant use NO | 216 | Reference | Reference | Reference |
| Anti-depressant use YES | 2796 | 1.37 (0.87-2.18) | 1.46 (0.92 -2.31) | 1.41 (0.88-2.24) |
| UPFs, Ultra-processed foods; PHQ-9, Patient Health Questionnaire-9.  *Fully adjusted model is adjusted for the following variables: age, sex, household income, education, ethnicity, born in Canada, smoking status, physical activity, daily alcohol consumption. | | | | |

**Supplementary Table 4** Results of cox regression for UPFs consumption and depression assessed using PHQ9 and anti-depressant joint association for incident type 2 diabetes in CARTaGENE

| **Model 1** **UPFs consumption lower & middle tertile combined and depressive symptoms joint association** | | | | |
| --- | --- | --- | --- | --- |
| **Groups** | **N** | **Unadjusted** | **Age- and Sex-Adjusted Model, HR (95% CI)** | **Fully Adjusted Model, HR (95% CI)** |
| LUND | 1789 | Reference | Reference | Reference |
| LUD | 219 | 1.07 (0.63 -1.84) | 1.19 (0.69-2.04) | 1.08 (0.63-1.87) |
| HUND | 866 | 1.25 (0.93 -1.68) | 1.27 (0.93 -1.73) | 1.24 (0.91 -1.67) |
| HUD | 138 | 1.62 (0.93-2.83) | 1.84 (1.05-3.217) | 1.67 (0.96 -2.97) |
| **Model 2 UPFs consumption lower & middle tertile combined and depressive symptoms/Antidepressant use joint association** | | | | |
| LUNDA | 1700 | Reference | Reference | Reference |
| LUDA | 308 | 1.27 (0.81-1.98) | 1.39 (0.89-2.16) | 1.30 (0.83-2.04) |
| HUNDA | 812 | 1.27 (0.93-1.74) | 1.29 (0.94-1.78) | 1.26 (0.91-1.74) |
| HUDA | 192 | 1.63 (1.00 -2.66) | 1.82 (1.11 -2.97) | 1.69 (1.03 -2.78) |
| LUND, lower/middle tertile of ultra-processed foods consumption and low depressive symptoms; LUD, lower/middle tertile of ultra-processed foods consumption and high depressive symptoms; HUND, higher tertile of ultra-processed foods consumption and low depressive symptoms; HUD, higher tertile of ultra-processed foods consumption and high depressive symptoms; LUNDA, lower and middle tertile of of ultra-processed foods consumption and low depressive symptoms and no antidepressant use; LUDA, lower and middle tertile of ultra-processed foods consumption and high depressive symptoms or antidepressant use; HUNDA, higher tertile of ultra-processed foods consumption and low depressive symptoms and no antidepressant; HUDA, higher tertile of ultra-processed foods consumption and high depressive symptoms or antidepressant.  *Fully adjusted model is adjusted for the following variables: age, sex, household income, education, ethnicity, born in Canada, smoking status, physical activity, daily alcohol consumption. | | | | |
